# Beverages

**Published:** 1874-08

**Authors:** 


### BEVERAGES.

MANY think that water does not answer all the purposes of slaking thirst, hence resort to what are called "Beverages," which mean liquids which contain some kind of stimulant. The habit of using these beverages is so insidious, and so uniformly leads to drunkenness, that every intelligent person ought to be able to know whether his favorite beverage is harming him or not, for he may become a drunkard before he knows it. All our habitual drinks are natural or unnatural—are beneficial and safe, or are injurious and dangerous. Good water is a natural drink, because the same amount of it quenches the thirst as well to-day as in childhood, and will do it as well when we get as old as Methuselah. And, generally, if you take a drink, you won't want any more for several hours, or perhaps until next day; and every time you take it, it quenches your thirst perfectly.

So it is as to healthful eating—you take the same number of slices of bread to-day as a year ago, or the same sized piece of meat at a meal, and you do not feel hungry until the next habitual meal. But all liquids which contain stimulants contain also the elements of drunkenness, from the mildest root beer to arack, brandy and absinthe, and are unnatural, because the same amount taken to-day as was taken a year ago will not give the same satisfaction it did a year ago. The same amount taken to-day will not give the same amount of satisfaction five years hence. You must increase the quantity, quality or frequency, or it does not answer the needs of the system. Therefore, whatever has to be increased in quantity or strength, to answer a given purpose, is an unnatural drink, and you use it at the peril of body, soul and estate.

